# Association between magnesium depletion score and prevalence of hyperuricemia in American adults: a study based on NHANES 2007-2018

**DOI:** 10.3389/fendo.2025.1438639

**Published:** 2025-02-06

**Authors:** Yongchun Xiao, Yong Mou, Ping Wu, Ke Wang, Tianjun Chen, Zhiwan Chen, Hongsheng Lin, Hua Yang, Zhisheng Ji

**Affiliations:** ^1^ Department of Orthopedics, The First Affiliated Hospital of Jinan University, Guangzhou, China; ^2^ Department of Microsurgery, The Affiliated Guangdong Second Provincial General Hospital of Jinan University, Guangzhou, China

**Keywords:** magnesium depletion score, hyperuricemia, uric acid, cross-sectional study, NHANES

## Abstract

**Background:**

The Magnesium Depletion Score (MDS) is a novel indicator that integrates multiple factors to assess systemic magnesium depletion. However, its association with hyperuricemia (HUA) prevalence remains unclear. This study aims to investigate the relationship between MDS and the prevalence of HUA.

**Methods:**

A cross-sectional analysis was conducted using data from the 2007-2018 National Health and Nutrition Examination Survey (NHANES). The MDS was derived by integrating multiple risk factors associated with magnesium depletion: the use of diuretics and proton pump inhibitors (PPI), estimated glomerular filtration rate (eGFR), and alcohol consumption exceeding recommended thresholds. Univariable and multivariable logistic regression models assessed the association between MDS and HUA. Subgroup and sensitivity analyses, including the exclusion of gout patients, further evaluated this association.

**Results:**

Among 18,761 participants, higher MDS were associated with an increased prevalence of HUA. Multivariable logistic regression confirmed a significant positive association between MDS and HUA (OR = 1.73, 95% CI = 1.62-1.84). Restricted cubic splines (RCS) further characterized the non-linear relationship between MDS and HUA prevalence. Subgroup analysis indicated a positive association between MDS and HUA, with significant interactions observed for sex and body mass index. Sensitivity and additional analyses reinforced the robustness of these findings.

**Conclusion:**

Among U.S. adults, higher MDS were significantly associated with an increased prevalence of HUA, suggesting that interventions targeting magnesium deficiency could be beneficial in reducing HUA prevalence within this population. However, prospective studies are needed to further confirm these findings.

## 1 Introduction

Hyperuricemia (HUA) is characterized as a chronic metabolic disease resulting from purine metabolism disorders (1), with elevated serum uric acid (SUA) levels as its primary clinical symptom. As the condition progresses, it may develop into gout, which leads to acute arthritis and tophi. Epidemiological studies estimate that about 38 million people reside in the US exhibit elevated SUA levels (2), especially among the obese population (3), with the prevalence of HUA rising annually (4). HUA serves as the primary risk factor for gout, an inflammatory arthritis (5); prolonged HUA may result in the formation of monosodium urate crystals (6), which damage joint structures and cause severe pain, significantly impacting a patient’s quality of life (7). Moreover, elevated SUA levels are linked to various diseases, including hypertension, kidney diseases, and cardiovascular diseases (8, 9). HUA has emerged as a serious public health issue, emphasizing the importance of research to evaluate factors associated with HUA.

Among the minerals found in the body, magnesium is the fourth most abundant cation and one of the important elements. It is essential for several physiological processes (10), such as energy metabolism, protein synthesis, and membrane integrity, among others (11, 12). A magnesium shortage can lead to alterations in biochemical pathways and is prevalent among obese patients (13), potentially increasing the risk of heart disease, diabetes, and other chronic conditions (14, 15). Current literature primarily focuses on serum or dietary magnesium, yet it does not adequately address the role of systemic magnesium deficiency in metabolic disorders, such as HUA. The conventional reliance on serum magnesium levels as an indicator of magnesium status may be insufficient, as these levels can remain within the normal range even during chronic deficiency, limiting their reliability in reflecting overall magnesium status. The Magnesium Deficiency Score (MDS) was initially proposed by Fan et al. in 2021 as a systematic scoring tool for assessing magnesium deficiency (16). A higher MDS indicates a greater degree of magnesium depletion. MDS aims to identify individuals with magnesium deficiency and encourage them to increase magnesium intake, thereby reducing systemic inflammation and maintaining stability in various biochemical and metabolic processes (17, 18). A significant relationship was found in a cross-sectional investigation among elevated MDS and a greater incidence of metabolic syndrome (19). The potential mechanisms linking MDS with HUA may involve inflammation and oxidative stress (20, 21). Additionally, low magnesium levels may affect DNA modification and repair, where damaged DNA and the ultimate breakdown of purine nucleotides can lead to increased uric acid production (22). However, studies are still lacking on the relationship between MDS and HUA. This study aims to address this gap by investigating the relationship between MDS and HUA in a nationally representative sample of U.S. adults, hypothesizing that a higher MDS is associated with an increased risk of HUA. Understanding this association may inform potential prevention strategies, underscoring the importance of magnesium status in metabolic health management.

## 2 Methods

### 2.1 Survey description

The National Health and Nutrition Examination Survey (NHANES), which is intended to assess the health and nutritional status of the American population, provided all information about participants for this research. This poll is cross-sectional and nationally representative. The Ethics Assessment Committee approved the study endeavor, and each participant provided signed informed permission.

### 2.2 Study population

Data for this study were collected from the NHANES between 2007 and 2018. Inclusion criteria included: (1) subjects aged 20 years or older and not pregnant at the time of examination; (2) participants with complete data on SUA concentrations and gout; (3) participants with comprehensive data necessary for assessing the MDS, including data on serum creatinine concentration, alcohol consumption, proton pump inhibitor (PPI) usage, and diuretic medication.

### 2.3 Diagnosis of HUA

SUA data for participants were obtained from the NHANES database. Blood samples from participants were collected, processed, and subsequently refrigerated or frozen in accordance with NHANES protocols. These samples were analyzed in laboratories designated by NHANES. SUA concentrations were measured using a standardized colorimetric method. HUA was defined as SUA levels above 7 mg/dL in males and 6 mg/dL in females. These thresholds, recommended by major clinical guidelines, are widely used in epidemiological studies to identify individuals at higher risk for HUA-related conditions (23, 24).

### 2.4 Calculation of MDS

The MDS is a scoring system developed based on previous studies to assess systemic magnesium depletion by integrating multiple risk factors associated with magnesium deficiency, including the use of diuretics and proton pump inhibitors (PPI), estimated glomerular filtration rate (eGFR), and alcohol intake exceeding recommended limits. MDS has shown predictive validity in identifying magnesium-related health risks, such as metabolic syndrome and systemic inflammation (16, 19). The score is calculated based on the following components: (1) one point for current diuretic use; (2) one point for current PPI use; (3) one point if eGFR is between 60 and 90 mL/min/1.73 m² and two points if eGFR is below 60 mL/min/1.73 m²; (4) one point for excessive alcohol intake, defined as more than two drinks per day for male and more than one drink per day for female. eGFR is calculated using the Chronic Kidney Disease Epidemiology Collaboration (CKD-EPI) formula. MDS range from 0 to 5, categorizing individuals into six groups: MDS = 0, 1, 2, 3, 4, and 5.

### 2.5 Covariates

The study’s main variables are chronic comorbidities and demographic traits. Age, race, ethnic origin, a person’s body mass index (BMI), poor index ratio (PIR), education level, smoking and drink alcohol, diabetes, hypertension, hypercholesterolemia and cardiovascular disease (CAD) are some of these factors. PIR is divided into three groups based on the poverty threshold: < 1, 1 - 3, and ≥ 3. It is computed by dividing the family’s earnings by this criterion. Individuals who have smoked in excess of 100 cigarettes in all of their lives are classified as smokers based on their answers to the questionnaire. Drinking status is defined as the consumption of at least 12 alcoholic beverages in a year. A history of CAD, heart failure that is congestive, or angina pectoris, or chest pain, is among the self-reported data from the questionnaire that is used to diagnose coronary heart disease. Other chronic comorbidities, including diabetes, hypertension and hypercholesterolemia, are identified through physician diagnoses or self-reports in the questionnaire.

### 2.6 Statistical analysis

In this study, data from the NHANES database, spanning six cycles from 2007 to 2018, were utilized. Following a filtering of the data according to participant inclusion criteria, the remaining participants were classified for statistical analysis according to their HUA status. Data that was categorical were reported as proportions, while continuous variables were shown as means ± deviations in standard form. Univariable and multivariable regression logistic analyses were carried out, yielding ratios of odds (OR) and 95% confidence intervals (CI), in order to investigate the association between the MDS and HUA. To explore the relationship between different MDS and HUA, and to illustrate the dose-response interaction, we used restricted cubic splines (RCS). The RCS model included three knots placed at the 10th, 50th, and 90th percentiles of the MDS distribution to capture potential non-linear relationships between the MDS and HUA. Subgroup analyses were conducted to assess the relationship between MDS and HUA across variables such as age, sex, smoking status, and the presence of various chronic illnesses. Sensitivity analyses were performed separately by excluding participants with gout and excluding participants with missing values for covariates. Three models were tested: Model 1 included no covariates; Model 2 adjusted for age, sex, and race; and Model 3 included additional adjustments for BMI, educational level, PIR, smoking, drink alcohol, hypertension, hypercholesterolemia, CAD and diabetes. All statistical analyses were performed using R (version 4.2.3), with a p-value < 0.05 considered indicative of statistical significance.

## 3 Results

### 3.1 Characteristics of study population

**Figure 1:**
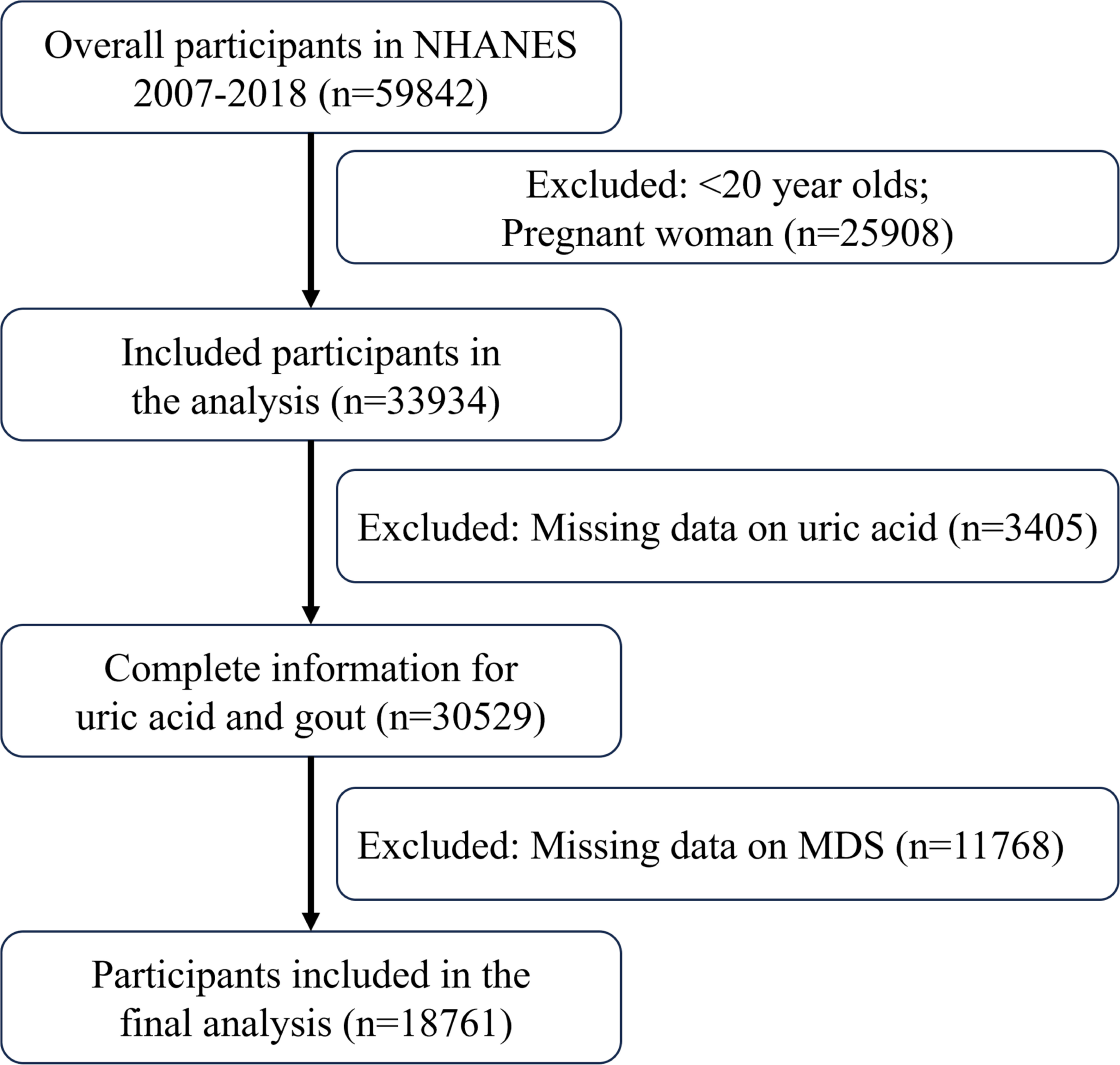
Include participants in the process.

Data were taken from the NHANES registry for 59842 participants between 2007 and 2018. The screening process is shown in **Figure 1**, with a final inclusion of 18,761 participants. **Supplementary Table 1** presents the demographic characteristics of the excluded population. **Table 1** displays initial traits based on the existence of HUA, whereas **Supplementary Table 2** displays weighted baseline features. Participants with HUA were generally older, male, and had higher BMI and education levels compared to those without the condition. Additionally, they exhibited higher SUA and serum creatinine (SCR) levels, coupled with lower eGFR. Additionally, there was a higher incidence of smoking in this group, as well as a higher chance of developing diseases including gout, diabetes, CAD, hypercholesterolemia and hypertension.

**Table 1:** Baseline characteristics of the study population.

Characteristic	Overall	Non-hyperuricemia	hyperuricemia	P-value
n	18761	15277	3484	
Age (%)				<0.001
<50	10666 (56.9)	9043 (59.2)	1623 (46.6)	
>50	8095 (43.1)	6234 (40.8)	1861 (53.4)	
Sex (%)				<0.001
Female	8685 (46.3)	7356 (48.2)	1329 (38.1)	
Male	10076 (53.7)	7921 (51.8)	2155 (61.9)	
Race (%)				<0.001
Mexican American	2768 (14.8)	2387 (15.6)	381 (10.9)	
Non-Hispanic black	3758 (20.0)	2900 (19.0)	858 (24.6)	
Non-Hispanic white	8330 (44.4)	6744 (44.1)	1586 (45.5)	
Others	3905 (20.8)	3246 (21.2)	659 (18.9)	
BMI (%)				<0.001
Underweight	271 (1.5)	259 (1.7)	12 (0.3)	
Normal	5173 (27.8)	4708 (31.0)	465 (13.5)	
Overweight	6249 (33.5)	5206 (34.3)	1043 (30.2)	
Obese	6934 (37.2)	5006 (33.0)	1928 (55.9)	
Education level (%)				0.004
Under high school	3613 (19.3)	2966 (19.4)	647 (18.6)	
High school or equivalent	4145 (22.1)	3296 (21.6)	849 (24.4)	
Above high school	10994 (58.6)	9007 (59.0)	1987 (57.0)	
No record	9 (0.0)	8 (0.1)	1 (0.0)	
PIR (%)				0.108
<1	3049 (17.8)	2507 (18.0)	542 (17.1)	
1-3	6866 (40.1)	5542 (39.8)	1324 (41.8)	
>3	7194 (42.0)	5889 (42.3)	1305 (41.2)	
Smoke (%)				<0.001
No	9583 (51.1)	7939 (52.0)	1644 (47.2)	
Yes	9164 (48.8)	7327 (48.0)	1837 (52.7)	
No record	14 (0.1)	11 (0.1)	3 (0.1)	
Drink alcohol (%)				0.31
No	1590 (8.5)	1317 (8.6)	273 (7.8)	
Yes	17164 (91.5)	13954 (91.3)	3210 (92.1)	
No record	7 (0.0)	6 (0.0)	1 (0.0)	
Hypertension (%)				<0.001
No	12507 (66.7)	10853 (71.0)	1654 (47.5)	
Yes	6235 (33.2)	4408 (28.9)	1827 (52.4)	
No record	19 (0.1)	16 (0.1)	3 (0.1)	
Hypercholesterolemia (%)				<0.001
No	10662 (63.5)	8896 (65.2)	1766 (55.8)	
Yes	6042 (36.0)	4671 (34.3)	1371 (43.3)	
No record	99 (0.6)	70 (0.5)	29 (0.9)	
Diabetes (%)				<0.001
No	16402 (87.4)	13535 (88.6)	2867 (82.3)	
Yes	1931 (10.3)	1430 (9.4)	501 (14.4)	
No record	428 (2.3)	312 (2.0)	116 (3.3)	
CAD (%)				<0.001
No	17689 (94.3)	14561 (95.3)	3128 (89.8)	
Yes	1072 (5.7)	716 (4.7)	356 (10.2)	
Hyperuricemia (%)				<0.001
No	15277 (81.4)	15277 (100.0)	0 (0.0)	
Yes	3484 (18.6)	0 (0.0)	3484 (100.0)	
Gout (%)				<0.001
No	17919 (95.6)	14829 (97.1)	3090 (88.8)	
Yes	828 (4.4)	439 (2.9)	389 (11.2)	
MDS (mean (SD))	1.19 (0.92)	1.08 (0.84)	1.68 (1.10)	<0.001
MDS (%)				
0	4019 (21.4)	3573 (23.4)	446 (12.8)	<0.001
1	9163 (48.8)	7906 (51.8)	1257 (36.1)	
2	3895 (20.8)	2901 (19.0)	994 (28.5)	
3	1311 (7.0)	745 (4.9)	566 (16.2)	
4	348 (1.9)	146 (1.0)	202 (5.8)	
5	25 (0.1)	6 (0.0)	19 (0.5)	
eGFR (mean (SD)) (ml/min/1.73m²)	92.95 (21.62)	95.42 (20.17)	82.11 (24.26)	<0.001
SCR (mean (SD)) (mg/dL)	0.89 (0.36)	0.86 (0.34)	1.03 (0.38)	<0.001
SUA (mean (SD)) (mg/dL)	5.52 (1.43)	5.04 (1.04)	7.60 (0.99)	<0.001

Mean ± SD for continuous variables, % for categorical variables. BMI, Body Mass Index; CAD, Coronary Artery Disease; MDS, Magnesium Depletion Score; PIR, Poverty Income Ratio; SCR, Serum Creatinine; SUA, Serum Uric Acid.

### 3.2 Association between MDS and HUA

**Figure 2:**
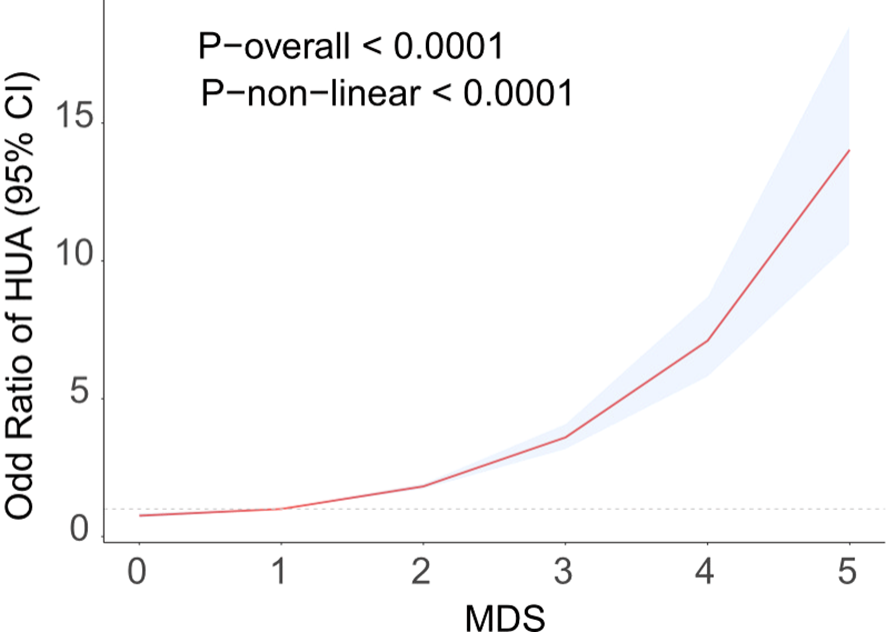
RCS curve fits the Association of MDS with Hyperuricemia. Adjusted for age, sex, BMI, race, educational level, PIR, smoking, drink alcohol, hypertension, hypercholesterolemia, CAD, diabetes.

**Table 2** displays the results of the logistic regression study evaluating the interaction among MDS and HUA. Univariate logistic regression analysis demonstrated a positive association between MDS and HUA (OR=1.85,95%CI=1.75-1.95). Multivariable adjustments, incorporating demographic and chronic disease covariates, sustained this association (OR=1.73,95%CI=1.62-1.84). When MDS was changed from a continuous to a categorical variable, high levels of MDS significantly increased the prevalence of HUA compared with low levels of MDS. RCS analysis further identified a nonlinear positive correlation between MDS and HUA prevalence, as shown in **Figure 2**. The prevalence of HUA gradually increased with increasing levels of MDS. The above results suggest that MDS maintains a robust positive association with the prevalence of HUA.

**Table 2:** The relationship between MDS and Hyperuricemia.

		Model 1 OR (95%CI) P-value	Model 2 OR (95%CI) P-value	Model 3 OR (95%CI) P-value
Hyperuricemia	MDS	1.85 (1.75, 1.95) <0.001	1.93 (1.82, 2.05) <0.001	1.73 (1.62, 1.84) <0.001
	0	[Reference]	[Reference]	[Reference]
	1	1.29 (1.10, 1.50) 0.002	1.36 (1.17, 1.58) <0.001	1.27 (1.06, 1.52) 0.011
	2	2.62 (2.25, 3.05) <0.001	2.50 (2.04, 3.07) <0.001	2.48 (2.06, 2.99) <0.001
	3	5.78 (4.73, 7.06) <0.001	6.77 (5.48, 8.36) <0.001	4.77 (3.73, 6.09) <0.001
	4	10.0 (7.48, 13.50) <0.001	12.7 (9.17, 17.60) <0.001	8.10 (5.75, 11.40) <0.001
	5	21.4 (6.90, 66.30) <0.001	24.0 (8.11, 71.20) <0.001	10.70 (2.91, 39.1) <0.001
	P for trend	<0.001	<0.001	<0.001

MDS, Magnesium Depletion Score; OR, Odds Ratio; CI, Confidence Interval; Model 1: No covariates adjusted; Model 2: Adjusted for age, sex, and race; Model 3: Adjusted for age, sex, BMI, race, educational level, PIR, smoking, drink alcohol, hypertension, hypercholesterolemia, CAD, diabetes.

### 3.3 Subgroup evaluation from MDS with HUA

**Figure 3:**
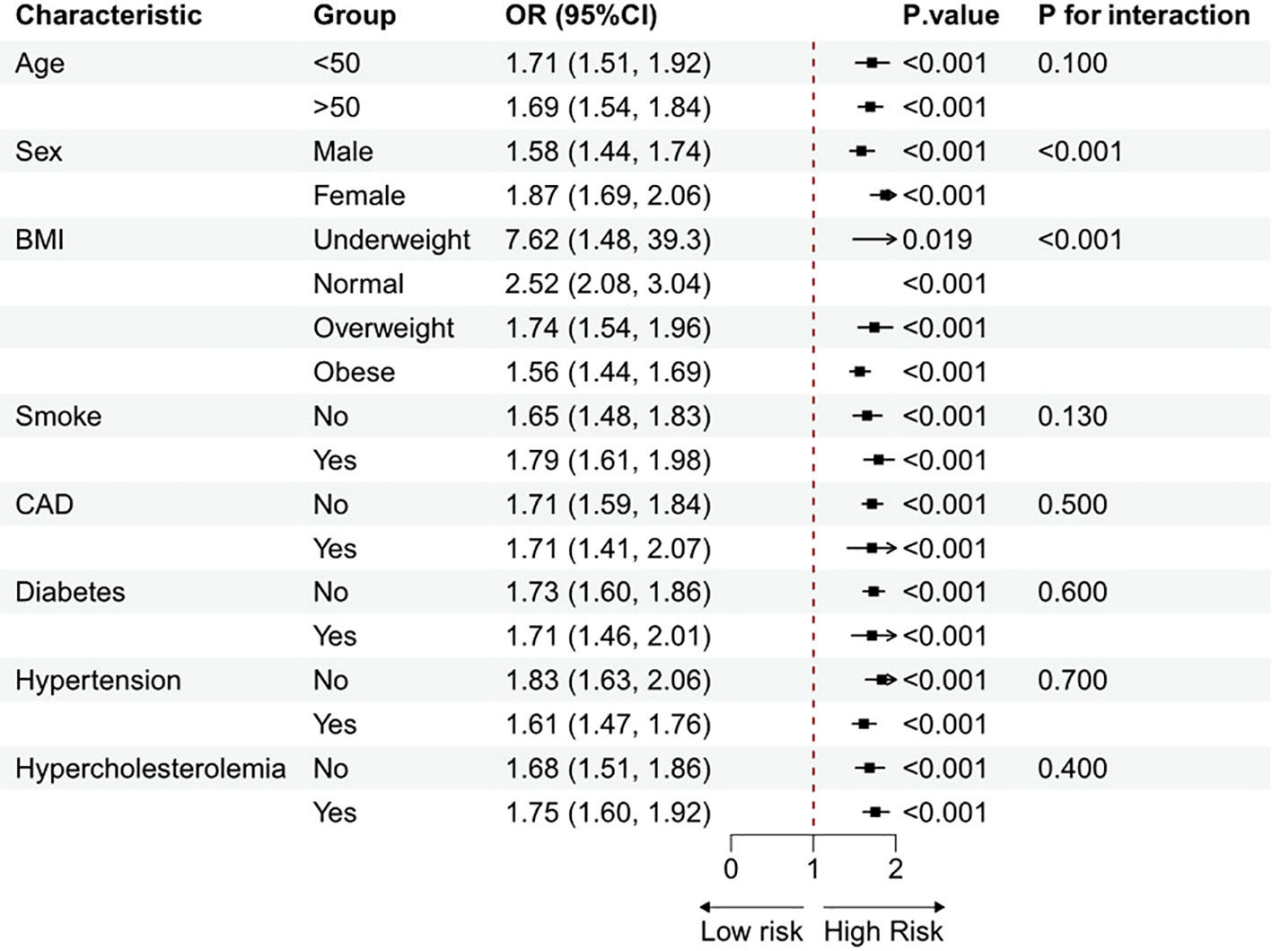
Subgroup analysis of the association between MDS and Hyperuricemia.

Subgroup analyses, stratified by age, sex, BMI, smoke, CAD, hypertension, diabetes, and hypercholesterolemia, were carried out to investigate any connections between MDS and HUA. **Figure 3** shows that a positive connection among MDS and HUA was found across certain subgroups after correcting for all variables. Interaction tests indicated potential influences on the correlation associated with sex and BMI. This positive correlation was particularly pronounced among females and participants with a lower BMI.

### 3.4 Sensitivity analysis

To rigorously evaluate the robustness of our findings, we performed a sensitivity analysis. Excluding 842 individuals with a gout diagnosis yielded a remaining sample of 17,919 participants. In this adjusted cohort, Model 3 logistic regression identified a significant positive association between MDS and HUA (**Supplementary Table 3**). Additionally, we excluded participants with missing covariate data, resulting in 16,216 participants available for further sensitivity analysis. The sensitivity analysis outcomes corroborated the primary study results, underscoring the robustness of the observed associations (**Supplementary Table 4**).

### 3.5 Additional analysis

Linear regression was employed in the additional analysis to investigate the relationship between MDS and SUA levels (**Supplementary Table 5**). The results indicated a stable positive correlation between MDS and SUA levels, whether MDS was considered a continuous or categorical variable. The RCS curve further demonstrated a nonlinear positive correlation between MDS and SUA levels (**Supplementary Figure 1**). These findings align with our analysis, enhancing the reliability of the relationship between MDS and HUA in this study.

## 4 Discussion

Using data from NHANES from 2007 to 2018, we investigated the relationship between MDS and HUA among adults in the United States. Our study found that participants with HUA were typically older, male, had a higher BMI, and also had higher MDS. A positive association between MDS and HUA prevalence was analyzed by logistic regression and was consistently confirmed in subgroup analyses and sensitivity tests. The above results suggest that MDS may be a potential indicator for assessing the prevalence of HUA.

Increasing evidence suggests that in addition to traditional factors such as genetics, high-purine diet, obesity, and metabolic syndrome, mineral intake also influences the risk of HUA (25, 26). Several studies indicate a correlation between serum magnesium levels and HUA (27, 28). Serum magnesium is the most commonly used method to assess magnesium status in clinical practice. Lower serum magnesium levels reflect higher MDS. Regarding the relationship between magnesium levels and HUA, existing studies have primarily focused on the effects of serum magnesium levels or dietary magnesium intake. For example, in India’s south, a study of 94 individuals with retinopathy caused by diabetes discovered an negative correlation between the patients’ blood magnesium concentration and urinary acid levels (29). Similarly, serum magnesium and HUA prevalence in males over 40 were shown to be inversely related in an observational study with 2,904 respondents. The study showed that, compared to the lowest quartile, the highest quartile had a 41% lower prevalence of HUA (OR = 0.59, 95% CI = 0.41-0.84) (28). However, because blood magnesium levels can stay within the usual range even during chronic insufficiency, they do not accurately reflect the underlying condition of the whole body magnesium (30, 31). Recent focus has shifted to dietary magnesium intake as a more accurate measure of magnesium status. A cross-sectional investigation of 5168 individuals over forty years of age old conducted in China found an adverse connection between HUA and magnesium from diet consumption (32). An further descriptive research comprising 26796 adult Americans found a correlation between a higher magnesium consumption and a lower incidence of HUA (29). Another way to measure magnesium status is by urine, which requires complicated 24-hour specimen gathering and is easily altered by food, diuretics, and renal function (30, 33, 34). The magnesium endurance test is complicated and challenging to extensively execute since it requires collecting 24-hour samples of urine, giving venous magnesium, and then collecting another 24-hour urinary sample. This test is frequently thought to be the best way to assess body magnesium levels (35). In contrast, this study selected MDS as a tool for systematically evaluating magnesium deficiency, as MDS provides a more comprehensive assessment of magnesium depletion by incorporating multiple factors, such as renal function and diuretic use, which addresses the limitations of traditional magnesium assessments (16). Based on population background information, men generally exhibit a higher prevalence of HUA, likely due to the protective effect of estrogen on uric acid metabolism in women (36). Additionally, men tend to consume alcohol and high-purine foods more frequently, both of which contribute to increased uric acid production (37). Conversely, patients with chronic diseases sometimes show lower uric acid levels, potentially due to medications like benzbromarone and allopurinol that promote uric acid excretion (38). The results of this study indicate a stable positive correlation between MDS and HUA, based on a broader MDS assessment, suggesting that MDS may better capture the role of magnesium in HUA risk than serum magnesium levels alone.

The potential mechanisms linking magnesium depletion and HUA may involve inflammation, oxidative stress, and metabolic disorders. Numerous studies have reported a positive correlation between HUA and inflammatory biomarkers, including CRP, TNF-α and N-methyl-D-aspartate, suggesting that uric acid may play a role in inflammation and subsequent inflammation-related diseases (39–41). Combined testing of serum uric acid and blood inflammatory markers may enhance the diagnostic accuracy for HUA (42, 43). Mechanistic studies have indicated a negative correlation between magnesium intake and CRP levels (44–48). Excessive serum uric acid crystallization can induce inflammation in joints and surrounding tissues (49), with patients experiencing uric acid crystals often showing markedly elevated serum CRP concentrations during acute episodes (50). Magnesium is essential for DNA folding and functional activity (22), with its role in DNA stability being concentration-dependent; low concentrations can lead to DNA deficiency and instability (51). Low magnesium levels may impact oxidative stress, subsequent oxidative DNA modifications, and DNA repair (52–54), potentially resulting in significant DNA damage and the release of purine nucleotides, which eventually degrade to produce uric acid (55). Given the association between HUA and oxidative stress and inflammatory cascades, the anti-inflammatory and antioxidant properties of magnesium may help mitigate these effects, providing biological plausibility for the positive correlation between MDS and HUA.

Our study has notable strengths, including its novelty and methodological rigor. It is the first to leverage a nationally representative sample to evaluate the association between HUA risk and the MDS. The findings demonstrate a consistent positive association between MDS and HUA, indicating that this relationship is unlikely due to random variation. Rigorous adjustments were made for demographic and chronic disease confounders, and stratified subgroup analyses were conducted to examine the MDS-HUA association across diverse demographic groups, highlighting the potential need for more targeted HUA prevention strategies. However, our study has several limitations. Firstly, the cross-sectional design of this study inherently limits causal inference; thus, future research incorporating *in vivo* or *in vitro* experimental validation is essential to substantiate these associations and clarify underlying mechanisms. Secondly, despite adjusting for a comprehensive set of covariates to assess the relationship between the MDS and HUA, unmeasured confounding variables may still have influenced the results, contributing to residual confounding. Finally, as a retrospective analysis, this study is susceptible to data collection biases, underscoring the need for prospective studies to confirm and reinforce the robustness of these findings.

## 5 Conclusions

Among U.S. adults, higher MDS were significantly associated with an increased prevalence of HUA, suggesting that interventions targeting magnesium deficiency could be beneficial in reducing HUA prevalence within this population. However, prospective studies are needed to further confirm these findings.

## Data Availability

Publicly available datasets were analyzed in this study. This data can be found here: https://www.cdc.gov/nchs/nhanes/nhanes.
