# Supplementary material for: Association between magnesium depletion score and prevalence of hyperuricemia in American adults: a study based on NHANES 2007-2018
Source: Front Endocrinol (Lausanne). 2025 Feb 6;16:1438639. doi: 10.3389/fendo.2025.1438639 (PMC11839436; doi:10.3389/fendo.2025.1438639)
Supplement: Supplementary file 1 [file DataSheet1.docx]

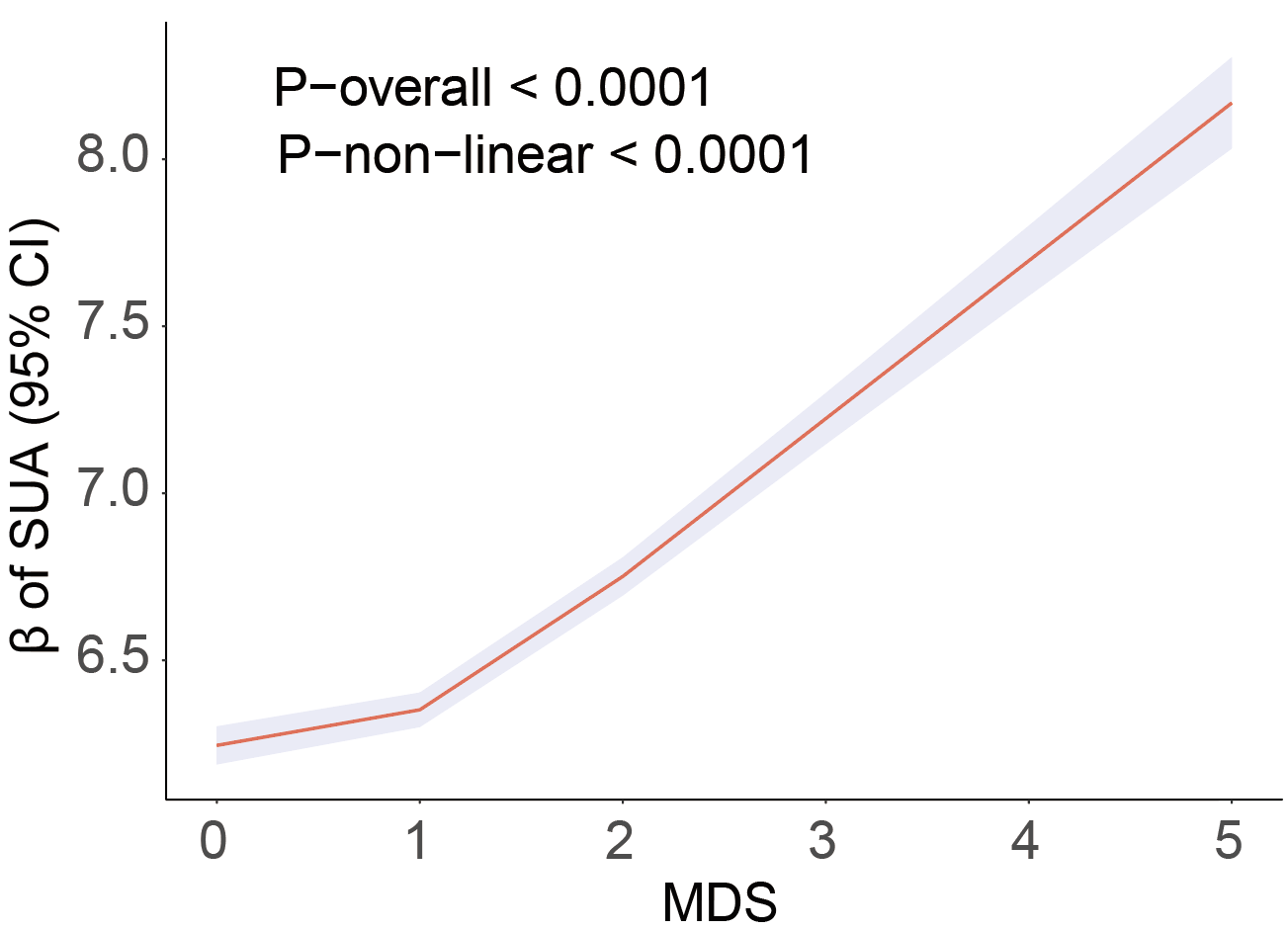


**Supplementary Figure 1 RCS curve fits the Association of MDS with SUA.**

Adjusted for age, sex, BMI, race, educational level, PIR, smoking, drink alcohol, hypertension, hypercholesterolemia, CAD diabetes.

**Supplementary Table 1: Weight Basic characteristics of the study population**

| Characteristic | Level | Overall |
| --- | --- | --- |
| n |  | 41081 |
| Age (%) | <50 | 29834 (77.3) |
|  | >50 | 8786 (22.7) |
| Sex (%) | Female | 21528 (52.4) |
|  | Male | 19553 (47.6) |
| Race (%) | Mexican American | 8146 (19.8) |
|  | Non-Hispanic black | 9604 (23.4) |
|  | Non-Hispanic white | 13068 (31.8) |
|  | Others | 10263 (25.0) |
| BMI (%) | Underweight | 10191 (29.9) |
|  | Normal | 10410 (30.6) |
|  | Overweight | 6515 (19.1) |
|  | Obese | 6948 (20.4) |

% for categorical variables. BMI: Body Mass Index

**Supplementary Table 2 Weight Basic characteristics of the study population**

| Characteristic | Overall | non-HUA | HUA | P-value |
| --- | --- | --- | --- | --- |
| n | 142863124.2 | 117803178.4 | 25059945.8 |  |
| Age (%) |  |  |  | <0.001 |
| <50 | 85445330.5 (59.8) | 72233398.5 (61.3) | 13211932.0 (52.7) |  |
| >50 | 57417793.7 (40.2) | 45569779.9 (38.7) | 11848013.8 (47.3) |  |
| Sex (%) |  |  |  | <0.001 |
| Female | 68619673.3 (48.0) | 59057445.6 (50.1) | 9562227.7 (38.2) |  |
| Male | 74243450.9 (52.0) | 58745732.8 (49.9) | 15497718.1 (61.8) |  |
| Race (%) |  |  |  | <0.001 |
| Mexican American | 11681045.4 (8.2) | 10090654.5 (8.6) | 1590390.9 (6.3) |  |
| Non-Hispanic black | 13979526.6 (9.8) | 11082551.2 (9.4) | 2896975.4 (11.6) |  |
| Non-Hispanic white | 99931540.8 (69.9) | 82145823.5 (69.7) | 17785717.2 (71.0) |  |
| Others | 17271011.4 (12.1) | 14484149.1 (12.3) | 2786862.3 (11.1) |  |
| BMI (%) |  |  |  | <0.001 |
| Underweight | 1972818.2 (1.4) | 1913317.0 (1.6) | 59501.2 (0.2) |  |
| Normal | 40942130.4 (28.8) | 37848673.5 (32.3) | 3093456.8 (12.5) |  |
| Overweight | 47266718.1 (33.3) | 39728228.6 (33.9) | 7538489.5 (30.4) |  |
| Obese | 51928870.7 (36.5) | 37793055.3 (32.2) | 14135815.3 (56.9) |  |
| Education level (%) |  |  |  | <0.001 |
| Under high school | 94840157.4 (66.4) | 78965869.4 (67.0) | 15874288.0 (63.3) |  |
| High school or equivalent | 30749104.8 (21.5) | 24606148.1 (20.9) | 6142956.7 (24.5) |  |
| Above high school | 39424.2 (0.0) | 38101.7 (0.0) | 1322.5 (0.0) |  |
| No record | 39424.2 (0.0) | 38101.7 (0.0) | 1322.5 (0.0) |  |
| PIR (%) | 17234437.8 (12.1) | 14193059.2 (12.0) | 3041378.6 (12.1) |  |
| <1 |  |  |  | 0.099 |
| 1-3 | 15214976.4 (11.5) | 12615532.3 (11.5) | 2599444.2 (11.2) |  |
| >3 | 44021322.6 (33.1) | 35827721.8 (32.7) | 8193600.8 (35.2) |  |
| Smoke (%) | 73609426.5 (55.4) | 61093778.3 (55.8) | 12515648.3 (53.7) |  |
| No |  |  |  | 0.001 |
| Yes | 75089577.6 (52.6) | 62774703.6 (53.3) | 12314874.0 (49.1) |  |
|  | 67709839.0 (47.4) | 54979782.6 (46.7) | 12730056.4 (50.8) |  |
| No record | 63707.7 (0.0) | 48692.2 (0.0) | 15015.5 (0.1) |  |
| Drink alcohol (%) |  |  |  | 0.239 |
| No | 9219797.5 (6.5) | 7728805.4 (6.6) | 1490992.0 (5.9) |  |
| Yes | 133566853.7 (93.5) | 110001506.7 (93.4) | 23565347.1 (94.0) |  |
| No record | 76473.0 (0.1) | 72866.3 (0.1) | 3606.7 (0.0) |  |
| Hypertension (%) |  |  |  | <0.001 |
| No | 99753525.8 (69.8) | 86667234.6 (73.6) | 13086291.2 (52.2) |  |
| Yes | 43002120.5 (30.1) | 31040189.6 (26.3) | 11961930.9 (47.7) |  |
| No record | 107477.9 (0.1) | 95754.2 (0.1) | 11723.8 (0.0) |  |
| Hypercholesterolemia (%) |  |  |  | <0.001 |
| No | 84472572.6 (64.7) | 71150581.8 (66.2) | 13321990.8 (57.7) |  |
| Yes | 45570361.7 (34.9) | 35987304.5 (33.5) | 9583057.2 (41.5) |  |
| No record | 544619.6 (0.4) | 372512.3 (0.3) | 172107.3 (0.7) |  |
| Diabetes (%) |  |  |  | <0.001 |
| No | 128709233.4 (90.1) | 107110492.7 (90.9) | 21598740.7 (86.2) |  |
| Yes | 11280368.6 (7.9) | 8533186.6 (7.2) | 2747182.0 (11.0) |  |
| No record | 2873522.2 (2.0) | 2159499.1 (1.8) | 714023.1 (2.8) |  |
| CAD (%) |  |  |  | <0.001 |
| No | 136137324.0 (95.3) | 113023762.9 (95.9) | 23113561.1 (92.2) |  |
| Yes | 6725800.2 (4.7) | 4779415.5 (4.1) | 1946384.7 (7.8) |  |
| Gout (%) |  |  |  | <0.001 |
| No | 137438594.6 (96.3) | 114801083.8 (97.5) | 22637510.8 (90.4) |  |
| Yes | 5346240.1 (3.7) | 2941408.7 (2.5) | 2404831.4 (9.6) |  |
| MDS (mean (SD)) | 1.14 (0.88) | 1.05 (0.81) | 1.56 (1.05) | <0.001 |
| MDS (%) |  |  |  | <0.001 |
| 0 | 31632147.5 (22.1) | 28154536.7 (23.9) | 3477610.8 (13.9) |  |
| 1 | 71517895.5 (50.1) | 61707580.2 (52.4) | 9810315.2 (39.1) |  |
| 2 | 29551610.9 (20.7) | 22321555.2 (18.9) | 7230055.7 (28.9) |  |
| 3 | 8010142.5 (5.6) | 4674736.7 (4.0) | 3335405.8 (13.3) |  |
| 4 | 2059085.8 (1.4) | 919442.0 (0.8) | 1139643.8 (4.5) |  |
| 5 | 92242.1 (0.1) | 25327.6 (0.0) | 66914.4 (0.3) |  |
| EGFR (mean (SD)) | 93.92 (20.15) | 95.82 (19.04) | 84.97 (22.68) | <0.001 |
| SCR (mean (SD)) | 0.88 (0.28) | 0.86 (0.27) | 0.99 (0.30) | <0.001 |
| SUA (mean (SD)) | 5.46 (1.40) | 5.03 (1.05) | 7.52 (0.93) | <0.001 |

Mean ± SD for continuous variables, % for categorical variables. BMI, Body Mass Index; CAD: Coronary Artery Disease; HUA: Hyperuricemia; MDS: Magnesium Depletion Score; PIR, Poverty Income Ratio; SCR: Serum Creatinine; SUA: Serum Uric Acid

**Supplementary Table 3 Sensitivity analysis between MDS and HUA in participants with excluded gout.**

|  |  | Model 1  OR (95%CI) P-value | Model 2  OR (95%CI) P-value | Model 3  OR (95%CI) P-value |
| --- | --- | --- | --- | --- |
| HUA | MDS | 1.83 (1.72, 1.95) <0.001 | 1.93 (1.80, 2.07) <0.001 | 1.75 (1.63, 1.89) <0.001 |
|  | 0 | [Reference] | [Reference] | [Reference] |
|  | 1 | 1.26 (1.08, 1.48) 0.005 | 1.33 (1.14, 1.56) <0.001 | 1.26 (1.04, 1.52) 0.019 |
|  | 2 | 2.47 (2.09, 2.92) <0.001 | 2.73 (2.29, 3.25) <0.001 | 2.45 (2.00, 2.99) <0.001 |
|  | 3 | 5.85 (4.72, 7.26) <0.001 | 7.06 (5.60, 8.91) <0.001 | 5.11 (3.88, 6.74) <0.001 |
|  | 4 | 9.80 (7.03, 13.7) <0.001 | 12.8 (8.83, 18.4) <0.001 | 8.49 (5.76, 12.5) <0.001 |
|  | 5 | 44.0 (8.03, 241) <0.001 | 53.3 (10.7, 265) <0.001 | 32.0 (4.89, 210) <0.001 |
|  | P for trend | <0.001 | <0.001 | <0.001 |

HUA: Hyperuricemia; MDS: Magnesium Depletion Score; OR: Odds Ratio; CI: Confidence Interval;

Model 1: No covariates adjusted

Model 2: Adjusted for age, sex, and race

Model 3: Adjusted for age, sex, BMI, race, educational level, PIR, smoking, drink alcohol, hypertension, hypercholesterolemia, CAD, diabetes.

**Supplementary Table 4 Sensitivity analyses between MDS and HUA in those with missing covariates excluded.**

|  |  | Model 1  OR (95%CI) P-value | Model 2  OR (95%CI) P-value | Model 3  OR (95%CI) P-value |
| --- | --- | --- | --- | --- |
| HUA | MDS | 1.84 (1.74, 1.94) <0.001 | 1.91 (1.80, 2.03) <0.001 | 1.73 (1.62, 1.84) <0.001 |
|  | 0 | [Reference] | [Reference] | [Reference] |
|  | 1 | 1.25 (1.06, 1.47) 0.009 | 1.32 (1.12, 1.55 (0.001 | 1.26 (1.05, 1.51) 0.014 |
|  | 2 | 2.59 (2.20, 3.05) <0.001 | 2.82 (2.37, 3.36) <0.001 | 2.49 (2.06, 3.02) <0.001 |
|  | 3 | 5.56 (4.51, 6.87) <0.001 | 6.42 (5.15, 8.00) <0.001 | 4.69 (3.65, 6.04) <0.001 |
|  | 4 | 9.40 (6.96, 12.7) <0.001 | 11.6 (8.34, 16.2) <0.001 | 8.15 (5.74, 11.6) <0.001 |
|  | 5 | 19.6 (6.52, 58.7) <0.001 | 22.0 (7.67, 63.0) <0.001 | 13.1 (3.99, 43.3) <0.001 |
|  | P for trend | <0.001 | <0.001 | <0.001 |

HUA: Hyperuricemia; MDS: Magnesium Depletion Score; OR: Odds Ratio; CI: Confidence Interval;

Model 1: No covariates adjusted

Model 2: Adjusted for age, sex, and race

Model 3: Adjusted for age, sex, BMI, race, educational level, PIR, smoking, drink alcohol, hypertension, hypercholesterolemia, CAD, diabetes.

**Supplementary Table 5 The relationship between MDS and SUA.**

|  |  | Model 1  β (95%CI) P-value | Model 2  β (95%CI) P-value | Model 3  β (95%CI) P-value |
| --- | --- | --- | --- | --- |
| SUA | MDS | 0.32 (0.29, 0.35) <0.001 | 0.39 (0.36, 0.41) <0.001 | 0.32 (0.30, 0.35) <0.001 |
|  | 0 | [Reference] | [Reference] | [Reference] |
|  | 1 | 0.06 (0.00, 0.13) 0.067 | 0.15 (0.10, 0.21) <0.001 | 0.12 (0.06, 0.17) <0.001 |
|  | 2 | 0.45 (0.38, 0.53) <0.001 | 0.58 (0.52, 0.65) <0.001 | 0.49 (0.43, 0.56) <0.001 |
|  | 3 | 1.00 (0.92, 1.20) <0.001 | 1.30 (1.10, 1.40) <0.001 | 1.00 (0.90, 1.20) <0.001 |
|  | 4 | 1.50 (1.30, 1.70) <0.001 | 1.80 (1.60, 2.00) <0.001 | 1.50 (1.30, 1.80) <0.001 |
|  | 5 | 2.00 (1.00, 2.90) <0.001 | 2.00 (1.20, 2.80) <0.001 | 1.60 (0.82, 2.50) <0.001 |
|  | P for trend | <0.001 | <0.001 | <0.001 |

CI: Confidence Interval; MDS: Magnesium Depletion Score; OR: Odds Ratio; SUA: Serum Uric Acid;

Model 1: No covariates adjusted

Model 2: Adjusted for age, sex, and race

Model 3: Adjusted for age, sex, BMI, race, educational level, PIR, smoking, drink alcohol, hypertension, hypercholesterolemia, CAD, diabetes.
